# CAGE-TSSchip: promoter-based expression profiling using the 5'-leading label of capped transcripts

**DOI:** 10.1186/gb-2007-8-3-r42

**Published:** 2007-03-26

**Authors:** Shintaro Katayama, Mutsumi Kanamori-Katayama, Kazumi Yamaguchi, Piero Carninci, Yoshihide Hayashizaki

**Affiliations:** 1 Laboratory for Genome Exploration Research Group, Genomic Sciences Center, RIKEN Yokohama Institute, Suehiro-cho, Tsurumi-ku, Yokohama 230-0045, Japan; 2 Bioinformatics Solutions Division, Nittetsu Hitachi Systems Engineering, Inc., Akashi-cho, Chuo-ku, Tokyo 104-6591, Japan; 3 Genome Science Laboratory, Discovery and Research Institute, RIKEN Wako Main Campus, Hirosawa, Wako 351-0198, Japan

## Abstract

Cap analysis gene expression (CAGE) technology has revealed numerous transcription start sites (TSSs) in mammals and has suggested complex promoter-based patterns of regulation. We developed the CAGE-TSSchip to detect promoter-based transcriptional activity. The CAGE-TSSchip is a customized oligonucleotide array that targets known TSSs identified by CAGE. A new labeling method, labeling capped transcripts from the 5'-end, had to be developed. The CAGE-TSSchip is accurate and sensitive, and represents the activity of each TSS.

## Abstract 2

A novel approach that combines CAGE expression analysis with oligonucleotide array technology allows for the accurate and sensitive detection of promoter-based transcriptional activity.

## Background

Many genome sequencing projects of model species are finished and a large number of full-length cDNAs have been isolated. Trends in large-scale life science are changing from collection of essential elements to developing an understanding of global biologic mechanisms. Transcriptional regulatory pathways are among the basal functional mechanisms that remain largely unknown; estimation of promoter activity is an essential component of analysis of regulatory networks. Large-scale analysis of the human and mouse transcriptomes using cap analysis gene expression (CAGE) technology [1], revealed numerous transcription start sites (TSSs) [2,3]. The TSSs are not randomly distributed; rather, they are concentrated at several short regions connected to each gene. On average there are five or more TSS clusters at one locus, and these are not only at the 5'-end of the gene but also within the open reading frame or 3'-untranslated region (UTR). Promoter-based expression clustering revealed that even TSS clusters in the same locus exhibit different expression patterns. This finding implies that the regulatory mechanism is defined by each TSS cluster. Measuring the transcriptional activity by using TSSs rather than genes would therefore lead to a better understanding of transcriptional regulatory mechanisms. Furthermore, promoter-based expression profiling is of benefit to the research community.

A tag-based approach for TSS analysis [4] such as CAGE requires deep sequencing when it is used to measure fluctuations in transcript expression, but deep sequencing is time consuming and expensive. Also, the various traditional expression profiling technologies did not represent the activity of each TSS but only the total activity of some TSSs. Searching among the microarray technologies for a technique that will permit large-scale promoter-by-promoter analysis, we modified our mature technology of purifying capped transcripts [5] and developed a new labeling method starting from the 5'-end of capped transcripts. This protocol made it possible for us to design an array for promoter-based expression profiling, which we named the CAGE-defined TSS chip (CAGE-TSSchip). We demonstrated its accuracy and sensitivity. Furthermore, by using CAGE-TSSchip we were able to predict principal regulatory factors.

## Results and discussion

### CAGE-TSSchip for mouse promoters

**Figure 1:**
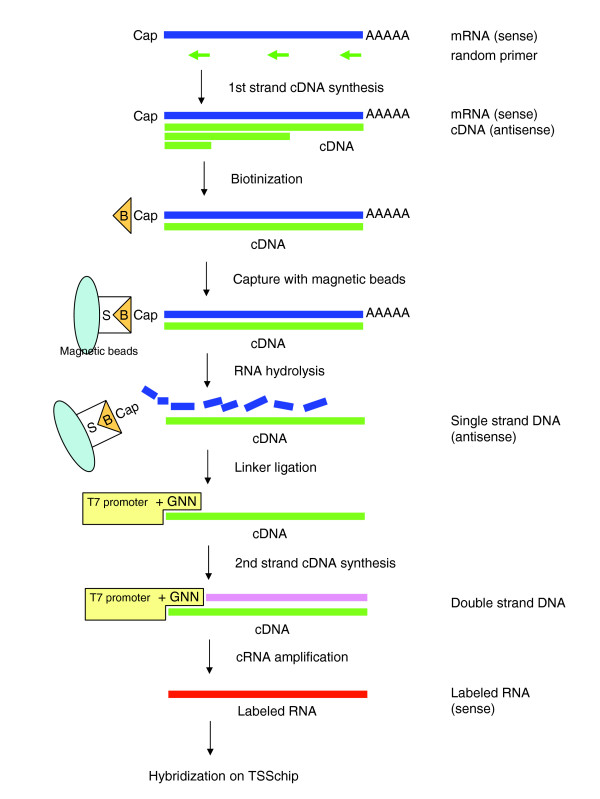
Schematic procedure of 5'-leading label of capped transcripts. The procedure is as described in more detail in Materials and methods (see text).

**Figure 2:**
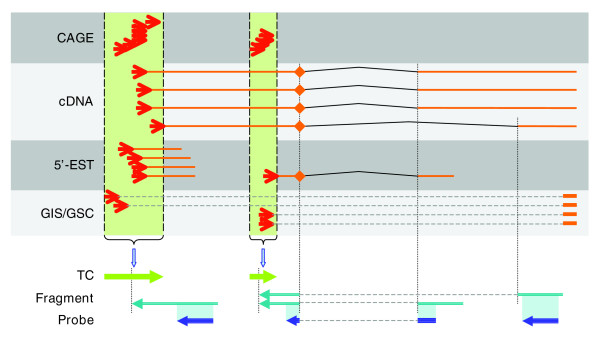
Overview of probe design: genomic coordination of TSSs and CAGE-TSSchip probes. The upper four tracks are an arrangement example of full-length transcripts (cDNA) and 5'-ends of transcripts derived from various methods (cap analysis gene expression [CAGE], 5'-expressed sequence tag [EST], and 5'-end of gene identification signature/gene signature cloning [4]). Tag clusters (TC; green arrow) are the overlapping regions of the 5'-ends. The most frequent transciption start site (TSS) for each TC is the representative position (vertical line from TC arrows). Fragments for the probe design, of 120-nucleotide long genomic sequences, starts from the representative position of each TC fragment, shown by cyan arrows. If the fragment overlaps the 5'-end of any exon-intron junction (diamond of cDNA and 5'-EST transcripts), the fragment skips the intron to the next exon. According to the Agilent probe design service, the 60-nucleotide appropriate region within each fragment would then be suggested for array probes (probe; blue arrows). Details of probe preparation are available in Additional data file 8.

Applying our technology to extraction of capped transcripts [6,7], labeling of the CAGE-TSSchip starts from the 5'-end of the capped transcripts (Figure 1). This is in contrast to traditional technology, in which labeling starts from the 3'-end of the transcript. Because it is difficult to transcribe labeled RNA from a certain downstream position to the cap site, we designed a linker containing a T7 promoter and ligated this linker to the 5'-end of the first strand full-length cDNAs. According to the sense of labeled RNAs, we spotted the antisense probes on the CAGE-TSSchip; this implies that the CAGE-TSSchip can identify the direction of transcription. Use of a tag-based probe design for promoter-based expression profiling, such as that proposed by Matsumura and coworkers [8], is not advisable because the distribution of TSSs affected by CpG islands is broad [2]. We therefore designed the CAGE-TSSchip probes to target the proximal regions of the promoters (Figure 2). We selected mainly transcription factors defined in TFdb [9], and extracted promoter sequences of these genes from the mouse CAGE database [10].

We isolated three total RNAs from mouse and conducted two comparisons using the CAGE-TSSchip; adult mouse liver versus mouse whole embryo in Theiler stage 17.5 (E17.5), and hepatocellular carcinoma cell line Hepa1-6 versus adult mouse healthy liver. We synthesized labeled RNAs using our 5'-leading method of capped transcripts and hybridized them to the CAGE-TSSchip. To estimate the reproducibility of our protocols, we designed dye swap experiments for these two comparisons. These experiments also helped us to reduce unavoidable technical variation [11]. After elimination of control, non-uniform, non-significant, or saturated spots, we deleted the hybridization signal that did not exhibit similar values in each dye swap experiment. The scatter plots for each dye swap experiment revealed good correlation (*r *= 0.87-0.96; Additional data file 2). The variation caused by procedures (described in Materials and methods, below) including our 5'-leading label method is therefore small.

### Accuracy and sensitivity: similar tendencies with qRT-PCR and CAGE

In order to confirm the accuracy of measurement of the expression ratio around promoters, we compared results with the CAGE-TSSchip with those with quantitative reverse transcription polymerase chain reaction (qRT-PCR). Even if the methods are different, it is important to demonstrate a similar tendency. First, we screened CAGE-TSSchip probes for which the ratio was threefold different or greater (absolute log ratio >0.5) between liver and E17.5. Then, we designed 20 qRT-PCR primers targeting similar regions of these probes (see Materials and methods, below). Table 1 summarizes findings with and comparison between CAGE-TSSchip and qRT-PCR. In all, 17 CAGE-TSSchip probes exhibited positive log ratios, which indicate high expression in the liver. Of these 17 probes, 16 showed similar positive log ratios to those for qRT-PCR measurements. Furthermore, there were three CAGE-TSSchip probes that exhibited similar negative log ratios to those of qRT-PCR measurements. Thus, the CAGE-TSSchip has an expression ratio similar to that of qRT-PCR.

**Table 1:** Cross-validation by qRT-PCR and CAGE in mouse liver versus E17.5

Target gene	CAGE-TSSchip		qRT-PCR	CAGE
	Probe ID	Ratio^a^	Ratio^a^	Ratio^a^
*Scp2*	pT04R06588376_1_55	1.54	2.22	2.25
*Phyh*	pT02F004A4350_1_56	1.47	1.48	2.09
*H2-Q7*	pT17F02067AF1_1_1	1.33	1.44	2.52
*Gcgr*	pT11F072A22AF_1_56	1.10	1.32	1.58
*1500017E21Rik*	pT19R022303AA_2_11	1.08	1.09	0.39^b^
*H2-K1*	pT17R01EFDD1A_2_55	1.08	1.36	3.04
*Ttr*	pT18F01417BA8_1_61	1.03	1.27	0.47^b^
*Creb3l3*	pT10R04D401D2_1_59	0.99	1.18	1.76
*Aldh7a1*	pT18R0366ABBD_1_60	0.95	0.39^b^	2.17
*Apoa1*	A_65_P16973as	0.93	1.14	0.76
*Ttr*	pT18F014197F5_1_51	0.93	1.14	0.56
*Ppara*	pT15F0521ABCF_1_61	0.92	0.92	1.77
*Bdh*	pT16F01DD69D0_1_61	0.83	0.91	0.93
*Hhex*	pT19F022F5EF6_1_61	0.75	0.88	1.63
*H2-K1*	pT17R01EF8909_1_61	0.67	-0.35^b,c^	2.89
*Trf*	A_65_P04625as	0.65	0.59	0.94
*Mdh1*	A_51_P218179as	0.57	0.19^b^	0.03^b^
*Mcm7*	pT05R08145E66_1_60	-0.52	-0.87	-0.33^b^
*Nisch*	pT14R019FAD28_1_56	-0.54	-0.27^b^	0.67^c^
*D0H4S114*	pT18R02055333_1_60	-0.67	-1.04	-0.95

Primer sequences are available in Additional data file 9. We assume that the absolute calue of the log ratio >0.5 is significantly different expression between liver and E17.5. ^a^Ratio = log_10_(liver/E17.5). ^b^Non-significance. ^c^Tendency for the sample to be highly different from the CAGE-TSSchip result. CAGE, cap analysis gene expression; qRT-PCR, quantitative reverse transcription polymerase chain reaction.

The frequency of CAGE tags reflects the activity of TSSs [2]. We examined whether this TSS activity shown by CAGE was reflected in the CAGE-TSSchip. We counted CAGE tag numbers in liver and E17.5 at the region upstream from the CAGE-TSSchip probe position (see Materials and methods, below). We focused on 20 probes, which once again were compared with qRT-PCR. In this comparison CAGE tags corresponding to 17 probes exhibited similar positive log ratios, and two of the three remaining probes exhibited similar negative log ratios (Table 1). Therefore, the CAGE-TSSchip also shows an expression ratio similar to the frequency identified by CAGE tag.

CAGE or similar serial analysis technologies require deep sequencing if they are to recognize fluctuations in weak promoter activity. Therefore, sensitivity is an important issue for the CAGE-TSSchip. To estimate sensitivity, we evaluated whether results with the CAGE-TSSchip and the corresponding qRT-PCR were similar even when promoter activity is low. First, we selected some CAGE-TSSchip probes, without considering the log ratio values in the liver versus E17.5 comparison, and designed 88 primers (see Materials and methods, below) corresponding to these probes. We then measured expression levels using qRT-PCR and compared expression ratios (Additional data file 3). In this comparison we could identify a tendency toward large mathematical error (difference) in the log ratio between the CAGE-TSSchip and qRT-PCR at high maximum qRT-PCR Ct value in liver and E17.5 (Additional data file 4a). These findings mean that the log ratios of rare transcripts tend to differ between the two methods. This is intuitive because such large Ct values in qRT-PCR, especially 30 or greater, also exhibit technical variations in repetitive experiments. In our experiments, the Ct value of 30 is equal to one transcript per eight cells. However, the log ratios in the liver versus E17.5 comparison were well correlated between CAGE-TSSchip and qRT-PCR (*r *= 0.77) in the 42 probes with a maximum Ct value above 30 (Additional data file 4b). About two million tags are required to recognize promoter-level fluctuations in expression of such rare transcripts (Ct value >30) when using CAGE; this imposes considerable burdens in terms of time and money. In conclusion, the CAGE-TSSchip is fast, has a good cost/performance ratio, and exhibits acceptable sensitivity.

### Observations: intensity of the CAGE TSSchip represents the activity of each TSS

**Figure 3:**
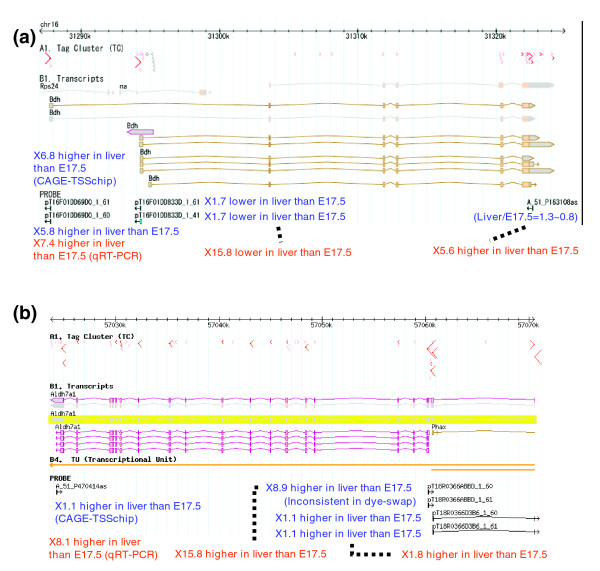
Observations of CAGE-TSSchip in liver versus E17.5 and genomic coordination. **(a) ***Bdh (Bdh1)*, which encodes 3-hydroxybutyrate dehydrogenase type 1. **(b) ***Aldh7a1*, which encodes aldehyde dehydrogenase 7 family, member A1. The red arrow in the tag cluster (TC) track describes cap analysis gene expression (CAGE) tag frequency, and TC width and direction. Transcript tracks show the splicing pattern and coding region of each transcript. PROBE shows the CAGE-TSSchip probes, and the blue values beside each probe are the intensity ratio between the liver and the E17.5 sample from the CAGE-TSSchip experiment. The red values on the bottom of each figure are the validated ratios, according to by quantitative reverse transcription polymerase chain reaction (also see Additional data file 5).

Having established the accuracy and sensitivity of the CAGE-TSSchip, we investigated several promoters of important genes in liver by comparing them between liver and E17.5. First, we focused on the liver-specific *Bdh *(*Bdh1*) gene, which encodes an enzyme (3-hydroxybutyrate dehydrogenase type 1) that is active in fatty acid metabolism and is an important marker of liver status. There are two isoforms in *Bdh*, and these isoforms do not share the first exons. The CAGE-TSSchip probe A_51_P163108as was designed based on the 3'-UTR of *Bdh *transcripts (Figure 3a and Additional data file 5). The intensities of liver and E17.5 were almost the same and were low (Additional data file 1). However, qRT-PCR clearly showed that *Bdh *expression was higher in liver than in E17.5. The CAGE-TSSchip probes pT16F01DD833D_1_61 and pT16F01DD833D_1_41 also targeted the first exon of the *Bdh*'s shorter isoforms, and for these probes the intensities were also low and almost the same between liver and E17.5. Although the result of qRT-PCR validation demonstrated a tendency toward lower expression in liver than in E17.5, there was considerable discrepancy in fold value. In contrast, the intensities of pT16F01DD69D0_1_61 and pT16F01DD69D0_1_60, targeting the first exon of *Bdh*'s longer isoforms, were clearly different. They were about 6.8-fold higher in liver than in E17.5. The qRT-PCR validation identified the same tendency and a similar fold value.

The discrepancy in minor promoters in liver between CAGE-TSSchip and qRT-PCR was expected because our labeling method involves the extraction of active promoters. The CAGE-TSSchip results also suggest that the regulatory mechanisms between these two promoters are different, even though they belong to the same gene. Findings of hierarchical clustering in CAGE expression [2] support this suggestion, because the expression patterns in that study were clearly different; the former promoter belongs to expression cluster number 4 and the latter to number 1 (Additional data file 6). Therefore, the CAGE-TSSchip findings in terms of these two isoforms are reasonable.

We then examined the *Aldh7a1 *gene, which encodes an important enzyme (aldehyde dehydrogenase 7 family, member A1) that is highly expressed in liver. As for *Bdh1*, there are two isoforms in *Aldh7a1*; however, the first exon of the short isoform shares the third exon of the long isoform. The CAGE-TSSchip has five probes for *Aldh7a1 *(Figure 3b and Additional data file 5). The CAGE-TSSchip findings suggest that the major promoter of *Aldh7a1 *in liver is the first exon of the short isoform; validation by qRT-PCR supports this finding.

Based on our design of the CAGE-TSSchip, we expected the intensity of CAGE-TSSchip findings to represent the activity of each TSS, which would lead to a considerably greater difference for 5'-side probes than for 3'-side ones. This tendency could be seen in *Bdh*, in *Aldh7a1*, and in other genes (for example *Ppp3ca*, *Scp2*, *Glo1*, *Fga*, and *Trf*). Because of this, we believe that the CAGE-TSSchip, as a tool for measuring TSS activity, will perform as we expected it to.

### E2F target genes were activated in Hepa1-6

We wished to demonstrate whether the CAGE-TSSchip makes it possible to analyze promoter-based regulatory mechanisms directly. It is noted that the functional regulatory elements that control transcription tend to be located close to TSSs [12]. We designed the CAGE-TSSchip probes at the proximal downstream region of known TSSs, and our protocols including the 5'-leading label method can demonstrate which TSSs are controlled. Because of this, the CAGE-TSSchip can help to identify important promoters and control elements. Below, we describe a comparison of Hepa1-6 and 'normal' adult mouse liver, and demonstrate both regulated (target) gene screening and regulator prediction.

In this comparison, 117 nonredundant probes of 98 genes identified over-expression (log ratio >0.5) in Hepa1-6, and 47 nonredundant probes of 36 genes revealed under-expression (log ratio <-0.5; Additional data file 7). In the comparison of the cancer cell line with normal tissue, many promoters of genes related to cell proliferation are expected to be extracted. Actually, genes related to DNA metabolism, which form the superclass of DNA replication in Gene Ontology (GO), were the most significantly enriched among the former genes (87/98 genes had some GO annotation and 21/87 genes were annotated with GO:0006259; *P *= 3.22 × e^-07 ^using GOstat [13]). Moreover, there were probes targeting the 5'-UTR or almost upstream of the genes encoding anaphase-promoting complex subunit 5 (*Anapc5*), minichromosome maintenance proteins (*MCM2-7*), and cyclin-dependent kinase 4 (*Cdk4*) in the former gene set. *MCM *genes have recently emerged as cancer biomarkers [14], and *Cdk4 *is important for cell cycle G1 phase progression. It is no surprise that abnormal proliferation occurs in the comparison between Hepa1-6 and 'normal' liver. Therefore, these target gene screen findings with CAGE-TSSchip agree well with current findings.

In order to identify regulatory factors of over-expressed genes in Hepa1-6, we estimated the over-represented transcription-factor binding sites (TFBSs) around the promoters of these genes. Table 2 shows the over-representation of the predicted TFBSs around the former probes in terms of Hepa1-6 over-expression promoters. The *E2F1 *binding site was the most over-represented TFBS. The probe for the *E2F1 *promoter also exhibited modest over-expression (log ratio about 0.43). Although the probe for the *Sp1 *transcription factor's promoter did not exhibit significant over-expression (log ratio about 0.27), the *Sp1 *binding site was over-represented. *Sp1 *is also related to cell growth and the cell cycle with phosphorylation events [15]. Kageyama and coworkers [16] suggested that the epidermal growth factor receptor (*EGFR*)-specific transcription factors (*ETF*) could also play a role in over-expression of the cellular oncogene *EGFR*. Therefore, we may conclude that regulator prediction using the CAGE-TSSchip is also reasonable.

**Table 2:** Over-represented TFBSs around the over-expressed promoter in Hepa1-6 compared with liver

TRANSFAC matrixID	|Log_10_(Hepa1-6/liver)| > 0.5				*P *value^a^	Binding factors
			
	Hepa1-6 > liver		Liver > Hepa1-6			
	Number of probes	%	Number of probes	%		
V$E2F1_Q3	87	74.4%	16	34.0%	2.31 × e^-06^	*E2f1*
V$SP1_01	73	62.4%	11	23.4%	6.04 × e^-06^	*Sp1*
V$ETF_Q6	62	53.0%	9	19.1%	9.96 × e^-05^	*ETF*^b^
V$ZF5_01	77	65.8%	16	34.0%	2.50 × e^-04^	*Zfp161*
V$CHCH_01	81	69.2%	18	38.3%	3.72 × e^-04^	*Churc1*

Total	117		47			

^a^Statistical significance of the over-representation of each transcription-factor binding site (TFBS) around the over-expressed promoter in Hepa1-6 compared with liver, determined using Fisher's exact probability test. ^b^Although TRANSFAC indicated that *EGFR*-specific transcription factor (*ETF*) binds to V$ETF_Q6 matrix, there was no report of this interaction in the mouse ortholog.

We note that the first exon of E2F transcription factor 7 (*E2F7*) was over expressed in Hepa1-6. As de Bruin and coworkers [17] pointed out, this gene could block the E2F-dependent activation of a subset of E2F target genes. *Zfp161 *and *Churc1 *are novel candidate regulators of Hepa1-6 over-expressed genes because the CAGE-TSSchip analysis revealed that these TFBSs are also over-represented around the Hepa1-6 over-expressed promoters. These novel regulators might represent an alternative regulatory pathway for Hepa1-6 phenotype.

We believe CAGE-TSSchip to be a useful tool in promoter-by-promoter analysis of regulatory networks. When similar prediction is performed using a non-promoter-specific microarray-based gene expression technology, representative transcripts (for example, RefSeq sets) are used to identify the genomic region that regulates promoter activity. The 5'-end of these representative transcripts is assumed to be the candidate TSS. Furthermore, proximal regions of these TSSs are candidate regulatory regions of transcription when this type of technology is used. If this traditional technology yields a similar result, then the regulated TSSs identified by the CAGE-TSSchip should overlap with the 5'-end of the RefSeq transcripts. However, out of the 163 TSSs belonging to over-expressed or under-expressed genes, 74 did not overlap with the 5'-end of the RefSeq sets. All of the cDNAs can be used to capture all of the TSSs; in this case, many unregulated TSSs would be included. For example, the probe set of the Affymetrix MG-U74 v2 chip could not define the singular TSSs in 26 out of the 124 genes that exhibited over-expressed or under-expression. Such probes show the summation of activities in all alternative promoters, and the search space for regulatory elements expands. Therefore, although the prediction of important regulators using the traditional expression profiling technology might be able to achieve similar results as the CAGE-TSSchip, one could assume that the significance would be lower. The CAGE-TSSchip has been optimized for promoter-by-promoter analysis.

## Conclusion

We developed the CAGE-TSSchip technology. This chip was able to identify the probes targeting the proximal region of the promoter defined by CAGE, and must be used with a new labeling method. This labeling method permitted labeling from the 5'-end of the capped transcripts. In a direct comparison between mouse liver and E17.5, CAGE-TSSchip identified expression ratios similar to those with qRT-PCR and CAGE, and had sufficient sensitivity to recognize the fluctuation in rare transcripts. Furthermore, the intensities of CAGE-TSSchip reflected the activity of each TSS, and so this technology may be useful in evaluating regulatory pathways.

CAGE-TSSchip permits promoter-based expression profiling with a favorable ratio of cost to performance and good accuracy by applying mature, two-color microarray technologies and equipment. Recently, several microarray platforms supporting one-color gene expression analysis for comparisons of many samples were developed. We were unfortunately unable to try these systems, but we will be able to change CAGE-TSSchip to a one-color analysis with minor modification.

In CAGE [1] and similar serial analysis technologies [4] for identification of novel TSSs, deep sequencing is necessary to identify promoters of rare transcripts or to compare expression levels in several samples. The current CAGE-TSSchip cannot identify novel promoters because we designed probes based on known transcripts and promoters, mainly defined by CAGE. However, several high-density microarray technologies will help us to identify novel promoters by combining them with our 5'-leading label method. A whole-genome tiling array is one approach to genome-wide promoter-based expression profiling.

An initial step in the analysis of transcriptional regulatory mechanisms is the identification of regulated elements and control elements. TSSs are just regulated elements, and major control elements are located around them. Therefore, promoter-based expression profiling is important in enhancing our understanding of regulatory mechanisms. CAGE-TSSchip and our 5'-leading label method is an alternative approach to promoter-based expression profiling, and it will help us to conduct promoter-by-promoter analysis of regulatory networks.

## Materials and methods

### Probe design

Figure 2 is an overview of the CAGE-TSSchip probe design. First, we defined tag clusters (TCs) from transcripts and several tag-based resources. Furthermore, we chose the representative position of a TC as the most frequent TSS. We selected about 4,500 TCs from about 2,500 transcriptional units [18], which were mainly transcription factors [9] as defined by CAGE tags from E17.5. We then prepared 120-nucleotide long genomic sequence fragments located in the proximity downstream of the representative position of the TC, according to splicing patterns of known transcripts. Custom Microarray Design Services (Agilent Technologies, Santa Clara, California, United States)) proposed appropriate 60-mer probes from each fragment. We adopted two (redundant) probes from each fragment, and added several control probes and reference probes (reverse complement to the Agilent Catalog Array probes; the prefix of the probeID is 'A_'). All probe sequences and their annotations are available in Additional data file 1, and details of the probe design are available in Additional data file 8.

### RNA preparation

Tissues from adult male and embryos from C57BL/6J mice were extracted according to the RIKEN Institute's guidelines. The Hepa1-6 cell line was purchased from the RIKEN Cell Bank (Tsukuba, Ibaraki, Japan) and was cultured in Dulbecco's modified eagle medium supplemented with 10% heat-inactivated fetal bovine serum, 200 U/ml penicillin, and 200 μg/ml streptomycin. The total RNA was extracted using the acid phenol guanidinium thiocyanate-chloroform method. Details of the RNA preparation are available in Additional data file 8.

### 5'-Leading label and hybridization

Figure 1 shows the schematic procedure of the 5'-leading label and hybridization process. The cDNA synthesis was performed using 50 μg of total RNA and with first-strand cDNA primer (random sequence) using SuperScript II RT (Invitrogen, Carlsbad, California, United States). The full-length cDNAs were then selected with the biotinylated cap-trapper method. A specific linker was used that contained the T7 promoter sites 'upper oligonucleotide GN3' (sequence 5'-ACTAATACGACTCACTATAGGNNN-3') and 'upper oligonucleotide GGN2' (sequence 5'-ACTAATACGACTCACTATAGGGNN-3'), which were mixed at a ratio of 4:1. This mixture was in turn mixed at a ratio of 1:1 to the 'lower oligonucleotides' (sequence 5'-TGATTATGCTGAGTGATATCC-3'). The sequence was then ligated to the single-strand cDNA. The second strand of the cDNA was synthesized with the T7 promoter primer and the DNA polymerase (TaKaRa, Ohtsu, Shiga, Japan). Details of cDNA synthesis, cRNA amplification for the 5'-leading label, and the hybridization are available in Additional data file 8.

### Quality check for the CAGE-TSSchip assay

Before analysis, control spots, saturated spots, non-uniform spots, and non-significant spots (according to Feature Extraction, the standard tool provided by Agilent for evaluating probe features) were removed. We also compared the Cy3 and Cy5 intensities of the same RNA samples in a dye swap experiment. We expected these signals to be correlated; however, low-intensity spots diverged somewhat from the regression line. We therefore excluded such probes that differed more than two times the standard residual deviation from the regression line. All intensity values and filtering results are available in Additional data file 1, and an overview can be found in Additional data file 2.

### Validation with qRT-PCR

Primer pairs were designed using an optimal primer size of 20 bases and annealing temperature of 60°C, using Primer3 software [19]. The uniqueness of the designed primers pairs was verified using the UCSC *in silico *PCR search in the UCSC Genome Browser Database [20]. This method checks that homologous regions are not cross-amplified by the same primer pair. All primers were also verified by amplification with mouse genome DNA. First-strand cDNA synthesis (5 μg total RNA per 20 μl reaction) was carried out using a random primer and the ThermoScript RT-PCR System (Invitrogen), in accordance with the manufacturer's protocol. A qRT-PCR was carried out with first-strand cDNA corresponding to 12.5 ng total RNA per reaction well using the tailor-made reaction [21]. The PCR reactions were performed with an ABI Prism (Applied Biosystems, Foster City, California, United States) using the following cycling protocols: 15 min hot start at 94°C, followed by 40 cycles of 15 s at 94°C, 30 s at 60°C, and 30 s at 72°C. The threshold cycle (Ct) value was calculated from amplification plots, in which the fluorescence signal detected was plotted against the PCR cycle. All primer sequences are available in Additional data files 3, 5 and 9.

### Validation with CAGE

Transcripts overlapping with probes serve as guides for the assignment between probes and CAGE tags. The total number of CAGE tags located from the probe position to 100 nucleotides upstream of the 5'-end of the overlapping transcripts is the expression level as estimated by CAGE. If several transcripts overlap with the same probe, then the transcript transcribed from the most upstream position is chosen as a representative transcript. CAGE tags are classified by RNA samples. The target RNA library IDs in this study were CBR, CCM and IN, corresponding to liver, Hepa1-6 and E17.5, respectively. Finally, log ratio values were calculated according to CAGE-TSSchip assays. Dataset details are available in Additional data file 8.

### Over-represented TFBS analysis

First, we chose probes exhibiting significant differences between Hepa1-6 and liver, with an absolute ratio above 0.5. After exclusion of redundant probes, we predicted the TFBSs around the probes in an area ranging from 1,000 nucleotides upstream to 200 nucleotides downstream using MATCH [22] from TRANSFAC [23] 9.4, with minimum false-negative profiles (minFN94.prf). The over-representation of each binding matrix was evaluated by using Fisher's exact probability test [24]. The matrices in Table 2 are the five most significantly over-represented ones in the regulatory regions of several genes, which exhibit higher expression in Hepa1-6 than in liver.

## Additional data files

The following additional data are available with the online version of this paper. Additional data file 1 provides TSSchip probe annotation and experimental results. Additional data file 2 shows the performance of 5'-leading label in dye swap experiments. Additional data file 3 provides details of sensitivity check with qRT-PCR. Additional data file 4 summarizes the sensitivity check with qRT-PCR. Additional data file 5 provides details of the alternative promoter check with qRT-PCR. Additional data file 6 provides CAGE expression clustering results of *Bdh *alternative promoters. Additional data file 7 summarizes over-expressed promoters in Hepa1-6 and liver. Additional data file 8 provides supplementary methods about the array probe design and whole protocols of wet experiments. Additional data file 9 gives details of cross-validation by qRT-PCR and CAGE in mouse liver versus E17.5.

## Supplementary Material

Additional data file 1 TSSchip probe annotation and experimental results. Some probe-sequences from the Agilent Catalog Array are not included in this data file because of a material transfer agreement between RIKEN and Agilent. (Please contact Agilent if you need these probe sequences.) Click here for file

Additional data file 2 Shown is the performance of 5'-leading label in dye swap experiments. Click here for file

Additional data file 3 Shown are the details of a sensitivity check with the qRT-PCR. Click here for file

Additional data file 4 Summarized is the sensitivity check with the qRT-PCR. Click here for file

Additional data file 5 Details of the alternative promoter check with qRT-PCR are given. Click here for file

Additional data file 6 Shown are CAGE expression clustering results of *Bdh *alternative promoters. Click here for file

Additional data file 7 Shown are over-expressed promoters in Hepa1-6 and liver Click here for file

Additional data file 8 Supplementary Methods regarding the array probe design and whole protocols of wet experiments are given. Click here for file

Additional data file 9 Shown are details of cross-validation by qRT-PCR and CAGE in mouse liver versus E17.5. Click here for file
