# Supplementary material for: CAGE-TSSchip: promoter-based expression profiling using the 5'-leading label of capped transcripts
Source: Genome Biol. 2007 Mar 26;8(3):R42. doi: 10.1186/gb-2007-8-3-r42 (PMC1868931; doi:10.1186/gb-2007-8-3-r42)
Supplement: Additional data file 2 — Shown is the performance of 5'-leading label in dye swap experiments. [file gb-2007-8-3-r42-S2.pdf]

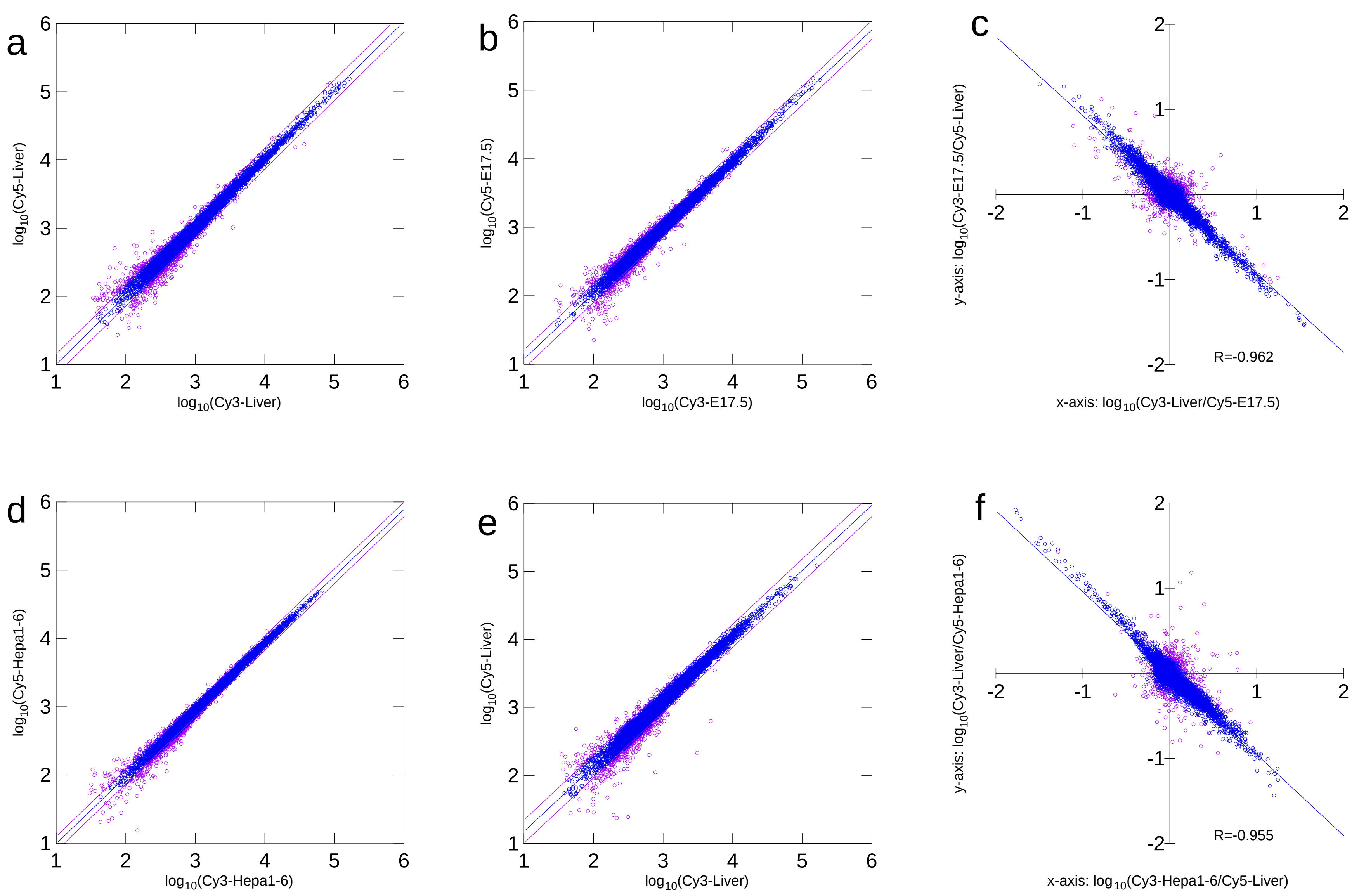

Additional data file 2; continued.

Additional data file 2: the performance of 5'-leading label in dye-swap experiments.

These are results of three experiments, adult mouse liver vs. mouse whole embryo in Theiler stage 17.5 (E17.5; a,b,c) and Hepa1-6 vs. liver (d,e,f). The four scatter plots (a,b,d,e) are comparison of the Cy3 and Cy5 intensities of same samples in each dye-swap; blue line is the regression line of all intensity points, and purple lines are two-times of standard residual deviation from the regression line. Blue points within the purple lines mean similar intensity in dye-swap. Allmost all of purpule points without purple lines are low intensities.
The other two scatter plots (c,f) are the correlation of dye-swap experiments. Blue points are reliable results calculated similar intensities (blue points of a,b,d,e) in dye-swap, and show the good correlation in each experiments. Purple points were excluded in the estimation of correlation coefficient.
