# Supplementary material for: CAGE-TSSchip: promoter-based expression profiling using the 5'-leading label of capped transcripts
Source: Genome Biol. 2007 Mar 26;8(3):R42. doi: 10.1186/gb-2007-8-3-r42 (PMC1868931; doi:10.1186/gb-2007-8-3-r42)
Supplement: Additional data file 4 — Summarized is the sensitivity check with the qRT-PCR. [file gb-2007-8-3-r42-S4.pdf]

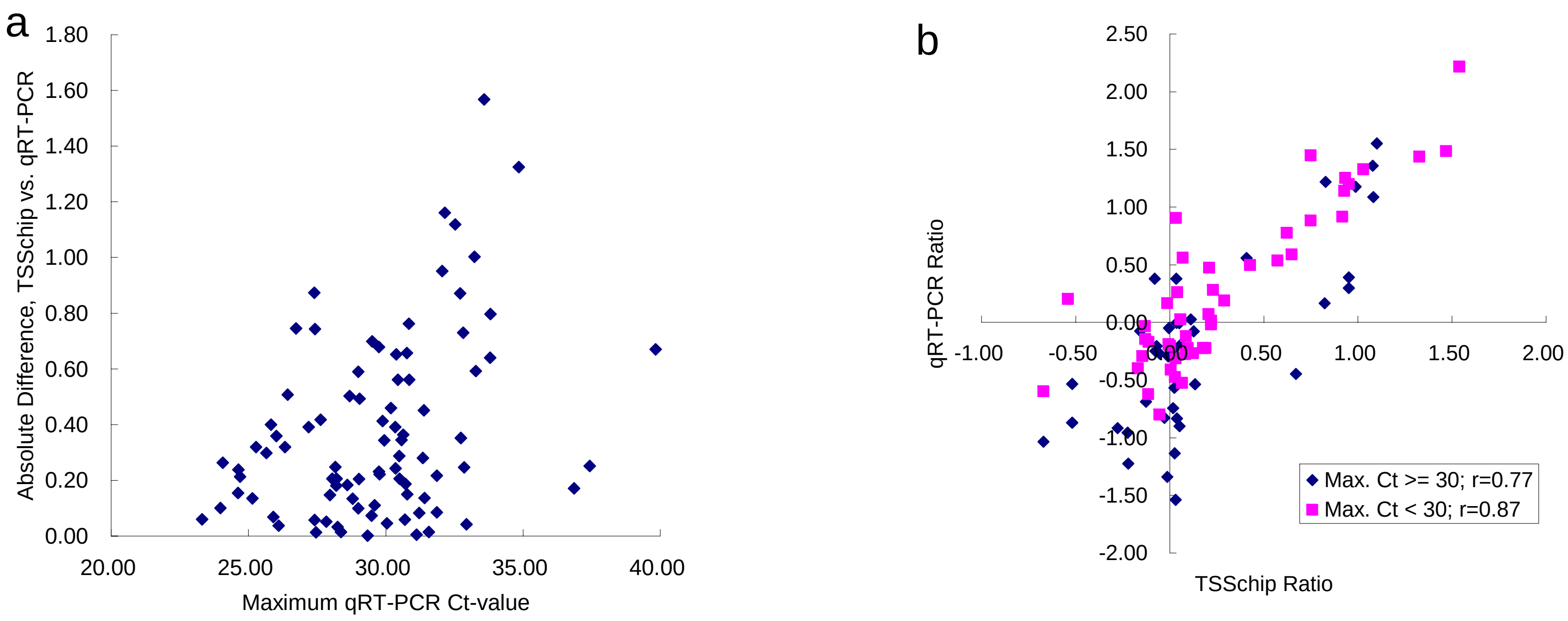

Additional data file 4: sensitivity check with the qRT-PCR

We performed comparison between liver and E17.5 to 88 probes. a) Scatter plot between the quantitative expression level and the error of the expression ratio. X-axis is the maximum Ct-value by the qRT-PCR in liver and E17.5. Y-axis is the difference of the expression ratio between the qRT-PCR and TSSchip. b) Correlation plot of the expression ratio between the qRT-PCR and TSSchip. X-axis is the expression log-ratio of the TSSchip. Y-axis is the expression log-ratio of qRT-PCR. Primer sequences and detail results are available in **Additional data file 3**.
