# Supplementary material for: CAGE-TSSchip: promoter-based expression profiling using the 5'-leading label of capped transcripts
Source: Genome Biol. 2007 Mar 26;8(3):R42. doi: 10.1186/gb-2007-8-3-r42 (PMC1868931; doi:10.1186/gb-2007-8-3-r42)
Supplement: Additional data file 6 — Shown are CAGE expression clustering results of Bdh alternative promoters. [file gb-2007-8-3-r42-S6.pdf]

Additional data file 6

a

b

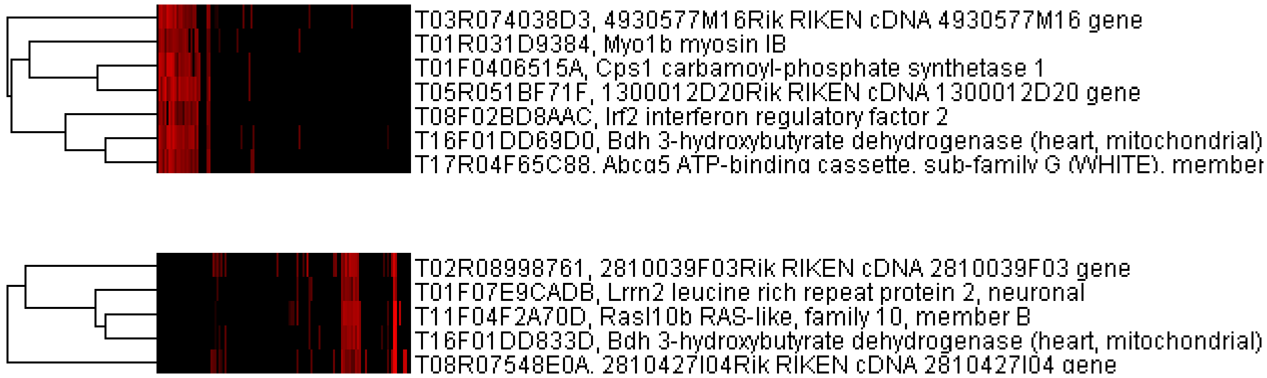

Additional data file 6: CAGE expression clustering results of Bdh alternative promoters

a) Expression pattern of *Bdh* first promoter (T16F01DD69D0) and the other correlated promoters at cluster 1.

b) Expression pattern of *Bdh* second promoter (T16F01DD833D) and the other correlated promoters at cluster 4.
