# Supplementary material for: CAGE-TSSchip: promoter-based expression profiling using the 5'-leading label of capped transcripts
Source: Genome Biol. 2007 Mar 26;8(3):R42. doi: 10.1186/gb-2007-8-3-r42 (PMC1868931; doi:10.1186/gb-2007-8-3-r42)
Supplement: Additional data file 8 — Supplementary Methods regarding the array probe design and whole protocols of wet experiments are given. [file gb-2007-8-3-r42-S8.doc]

**Katayama et al. TSSchip: the promoter-based expression profiling using 5’-leading label of capped transcripts.**

**Additional data file 8: SUPPLEMENTARY METHODS**

**Probe design**

Figure 2 is overview of the probe design. First we defined the promoters with same way of Tag Cluster in[3]. The data set of transcripts and tags are also same with[3]; there are 102,597 FANTOM3 cDNA sequences, 56,006 GenBank[25] mouse mRNA sequences (Release 139.0 + daily Jan.27, 2004), 25,803 RefSeq[26] mouse sequences (NM and NR at cumulative Jan.25, 2004, XM and XR at Sep.1, 2004), 35,247 EMSEMBL[27] mouse sequences (Release 25.33a), 11,567,973 CAGE[1] mouse tags, 385,797 mouse GIS di-tags, 2,079,652 mouse GSC di-tags[4] and 558,686 RIKEN 5’-EST. The transcripts and ESTs were mapped to the UCSC mm5 assembly[28] using Blat[29] and Sim4[30]. The tags were mapped to the same assembly using BLAST[31]. Overlapping region of CAGE tags, 5’-end of ditags and 20nt in 5’-end of transcripts in same strand is TC. Representative position of TC is the most frequent starting site in CAGE; if there was no CAGE tags, GIS, GSC, RIKEN 5’-EST and FANTOM3 transcripts are sequentially referred to define the representative position. The most upstream-position is the representative if there were the same tag numbers in two or more starting-sites. After the definition of promoter and their representative position, we cut out the 120nt genomic sequences starting from representative position of each TC. If such fragment overlaps to any introns of known transcripts, we eliminated such intron region and concatenate the next exon region; in other words, according to the splicing patterns of overlapping transcripts, there were two or more fragments starting from same transcription start site. These fragments were turned reverse-complementally, and formatted as “Sequence Submission Guidelines” of Agilent. Agilent selected the appropriate 60-mer probes from each 120nt fragments. All probe sequences and their annotations are available in **Additional data file 1.**

**RNA preparation**

We may use the basic market kit for purification of total RNA or extract total RNA by the acid phenol guanidinium thiocyanate-chloroform method[32]. But total RNA is recommended to be prepared from various tissues by modification of a standard procedure with adaptation of the CATB (cetyltriammonium bromide) precipitation method for selective removal of polysaccharides. This protocol has also has been used for cultured cells. In the presence of polysaccharides, the binding of full-length cDNA to streptavidin-coated magnetic beads is inhibited, perhaps by competing for the available streptavidin sites. We describe here the method of how to remove the polysaccharides and to refine RNA.

***Removing of polysaccharides***

1. Remove the polysaccharides from the RNA preparation by precipitation with CTAB solution. Add 4 volume of CTAB solution (1 % CTAB, 4 M Urea, 50 mM Tris-HCl (pH 7.0), 1 mM EDTA (pH 8.0)) and 1.3 volume of 5M NaCl, to the solution of RNA.
2. Recover the RNA by centrifugation at 9,500g for 15 minutes at room temperature (20-24ºC).
3. Discard the supernatant and resuspend the RNA pellet in 4 ml of 7 M guanidinium chloride.
4. Add 8 ml of ethanol (99 %) to the RNA solution. Mix the solution well and store it for 1-2 hours at -20 °C.
5. Recover the RNA by centrifugation at 9,500g for 15 minutes at 4 °C.
6. Wash the pellet of RNA in 80 % ethanol and re-centrifuge.
7. Re-dissolve the RNA in 100 μl to 1 ml of H2O.

**First-strand cDNA synthesis**

1. To prepare the first-strand cDNA, put together the following reagents in 0.5ml tube:
   - Total RNA (50 μg)
   - Random primer [2 μg/μl] 2.5 μl (5 μg)
   - H2O (sterilized distilled water, to a final volume of 55 μl)
2. Heat the mixture of RNA and primers to 65 °C for 10 minutes and put on ice for 5 minutes to melt secondary structures in the RNA.
   - 5X 1st strand buffer 20 μl
   - 10 mM dNTP solution 5 μl
   - 0.1 M DTT 10 μl
   - RNase OUT [40 U/μl] 5 μl
   - RNase H-SUPERSCRIPT II RT [200 units/μl] 5 μl
3. Incubate for 1 hr at 42 °C.

**Purification of first-strand cDNA by digestion with proteinase K**

1. Add 2 μl of proteinase K [20 μg/μl]. Incubate the reaction for 15 minutes at 45 °C
2. Add 400 μl of CTAB solution and 40 μl of 5M NaCl. Mix and incubate for 10 minutes at room temperature. And then centrifuge this at maximum speed for 15 minutes at room temperature. Discard the supernatant and resuspend the pellet in 200 μl of 7M Guanidin-HCl. Add 500 μl of ethanol (99 %), mix and store at -20 °C for more than 30 minutes.
3. Centrifuge the mixture at 15,000g for 20 minutes at 4 °C and remove the supernatant. Very gently wash the pellet twice with 750 μl of 80 % ethanol. Centrifuge at 15,000g for 5 minutes at 4 °C after each wash for 3 minutes.
4. Dissolve the cDNA in 44.7 μl of H2O (OBS! Do not use solutions containing Tris or other buffers with polyhydroxy groups that can be oxidized to diols).

**Oxidation and biotinylation of diol groups of mRNA**

In this protocol, the diol groups of RNA (the cap structure and the 3' terminus) are first oxidized in the presence of sodium iodate and subsequently coupled with the biotin hydrazide long arm variant (also called biocytin hydrazide). Biotinylated cDNA-RNA hybrids may then be isolated using streptavidin-coated beads as described later.

***Oxidation***

1. Add the following reagents directly to the 44.7 μl derived from the reaction described above of the first-strand cDNA.
   - 3.3 μl of 1 M sodium acetate (pH 4.5) to a final concentration of 66 mM
   - 2 μl of 250 mM NaIO4 (freshly prepared) to a final concentration of 10 mM
2. Incubate the mixture on ice in the dark for 45 minutes.
3. Stop the reaction by adding 1 μ1 of 80 % glycerol and mixing, for glycerol to react with NaIO4 to quench the reaction.
4. Precipitate cDNA/RNA with 100 μ1 of ethanol (99 %), 0.5 μl of 10 % SDS, and 2.5 μl of 5 M NaCl. Incubate the mixture for more than 30 minutes at -20 °C and then centrifuge in 4 °C at 15,000g for 20 minutes.
5. Wash the pellet in 200 μ1 of 80 % ethanol and centrifuge (4 °C) at 15,000g for 5 minutes. Carefully discard the supernatant.
6. Centrifuge again briefly and remove any remaining ethanol. Dissolve the nucleic acids in 50 μ1 of H2O.

*Biotinylation*

1. Add 5 μl of 1 M sodium citrate (pH 6.1), 5 μl of 10 % of SDS and 150 μl of a freshly prepared solution of 10 mM biotin hydrazine (long arm variant) to oxidated cDNA/RNA (50 μ1) obtained in Step 6. Mix the solution gently and then incubate it overnight (10-16 hours) at room temperature (20-24°C).
2. At the end of the incubation, precipitate the biotinylated cDNA-mRNA hybrid by adding the following. After gentle mixing, incubate the ethanolic solution for more than 30 minutes at -20 °C.
   - 1 M Na acetate (pH 6.1) 75 μ1
   - 5 M NaCl 5 μl
   - ethanol (99 %) 525 μl
3. Recover the precipitate by centrifuging the sample at 15,000g for 20 minutes at 4 °C. Discard the supernatant and remove any free biotin hydrazide from the pellet by washing twice with 80 % ethanol, centrifuging at 4 °C after each wash.
4. Dissolve the final pellet in 179 μl of H2O.

**CAP-trapping and release of full-length cDNA**

***Digestion with RNase***

1. Add the following to the cDNA sample (180 μl)
   - 10X RNase I buffer (Promega) 20 μl
   - RNase I [10 units/μl] 1 μl
2. Incubate the reaction mixture for 30 minutes at 37 °C.
3. After stopping the reaction, add the following to the reaction:
   - SDS (10 %) 4 μ1
   - proteinase K (20 μg/μl) 2 μl
4. Incubate the reaction mixture for 15 minutes at 45 °C.
5. After proteinase K treatment, extract the solution with phenol:chloroform and then with chloroform. Back-extract (transfer the upper phase to a new tube) the organic phase with H2O and precipitate the nucleic acids using ethanol (99 %) and 3.2 μl of 10 μg/μl tRNA as carrier. Add 20 μl of 5 M NaCl and 800 μl of ethanol (99 %), and store for 30 minutes at -20 °C. Recover the precipitate by centrifuging the sample at 15,000g for 20 minutes at 4 °C and wash the pellet twice with ethanol (80 %). Dissolve the pellet in 50 μl of H2O.

***Pretreatment of Magnetic Beads***

1. Pretreat 500 μl of MPG beads (500 μl per 50 μg of starting total RNA) with 50 μg of DNA-free tRNA (10 μg/μl of yeast tRNA). Incubate the beads for 30 minutes on ice with occasional mixing to prevent bead sedimentation.
2. Separate the beads by standing the tube in a magnetic rack for a few minutes. Carefully remove the supernatant and wash the beads three times with 500 μ1 of binding buffer (4.5 M NaCl, 50 mM EDTA, pH8.0).
3. Resuspend in 500 μl of 4.5 M NaCl, 50 mM EDTA, pH8.0.

***Capture of Full-length cDNA***

1. Add 350 μl the tRNA-treated MPG beads (step 8) to a fresh tube containing the biotinylated first-strand cDNA obtained at Step 4 (50 μ1 of cDNA in H2O). After pipetting, incubate the reaction for 10 minutes at 50 °C, with slow end-over-end rotation.
2. At the end of the incubation, transfer the remaining 150 μl of beads to the reaction tube and continue the incubation for a further 20 minutes at 50 °C.
3. Separate the beads by standing the tube in a magnetic rack for few minutes.
4. Carefully remove the supernatant and wash the beads as follows (wash volume is 0.5 ml):
   - 2X with washing/binding solution (4.5 M NaCl, 50 mM EDTA, pH 8.0)
   - lX with 0.3 M NaCI/1 mM EDTA
   - 3X with 0.4 % SDS/0.5 M sodium acetate/20 mM Tris-HCl, pH 8.5/1 mM EDTA
   - 2X with 0.5 M sodium acetate/l0 mM Tris-HCI, pH 8.5/1 mM EDTA

***Eluting the cDNA***

1. After the final wash, elute the cDNA by adding 100 μ1 of 50 mM NaOH/5 mM EDTA. Stir the beads briefly and rotate them for 5 minutes at room temperature with vortex and tapping.
2. Separate the magnetic beads and transfer the supernatant (containing the eluted cDNA) to a fresh tube. Store the cDNA on ice.
3. Repeat the elution twice more, each time using 100 μ1 of 50 mM NaOH/5 mM EDTA.
4. Store the pooled elutes on ice to prevent any hybridization between cDNAs and any contaminating RNAs that might be present.
5. Add 60 μ1 of 1 M Tris-Cl (pH 7.0) to each tube (on ice) and mix quickly.
6. Add an equal volume of phenol:chloroform. Mix the organic and aqueous phases and then centrifuge the emulsion at maximum speed for 5 minutes at room temperature in a microfuge. Transfer the aqueous upper layer to a fresh tube.
7. Precipitate cDNAs from the supernatant by adding 30 μl of 5 M NaCl and 600 μl of isopropanol at -20 °C (and add 2 μl of glycogen as carrier).
8. Collect the cDNAs by centrifugation at maximum speed for 20 minutes at 4 °C in a microfuge. Remove the supernatant and any drops of fluid adhering to the walls of the tube.
9. Add 500 μl of 80 % ethanol and recover the cDNA by centrifugation at maximum speed for 5 minutes at 4 °C in a microfuge. And wash the pellet twice with 80 % ethanol.
10. Remove the supernatant and dissolve cDNA in 40 μl of H2O.
11. Apply the sample to the column (S300 spin column by Amersham-Biosciences) and centrifuge at 700g for 2 minutes in room temperature. Apply 40 μl of H2O and repeat the centrifugation.
12. Add 80 μl of 5 M ammonium acetate (pH5.2) and 200ul of ethanol (99 %), and store for more than 30 minutes at -20 °C.
13. Centrifuge the mixture at 15,000g for 20 minutes at 4 °C and remove the supernatant. Very gently wash the pellet twice with 500 μl of 80 % ethanol. Centrifuge at 15,000g (4 °C) after each wash for 5 minutes.
14. Dissolve the cDNA in 2 μl of H2O.
15. Heat the 0.01-0.1 pmol cDNA solution for 5 minutes at 65 °C and transfer the tube onto ice.

**Adding a priming site to the 5’ end of first-strand cDNA**

1. Mix 1.5 μl of 0.4 μg/μl linker mixture and 3.5 μl of H2O. Linkers (pair of linker/pair 1 and 2) has been adjusted beforehand as double-stranded DNA.
   - linker mixture (linker 1 : linker 2 = 1 : 4);
     - linker 1 / pair 1 (sequence: ACTAATACGACTCACTATAGGNNN)
     - linker 1 / pair 2 (sequence: TGATTATGCTGAGTGATATCC)
     - linker 2 / pair 1 (sequence: ACTAATACGACTCACTATAGGGNN)
     - linker 2 / pair 2 (sequence: TGATTATGCTGAGTGATATCC)
2. Incubate at 37 °C for 5 minutes and transfer onto ice.
3. Add 2.5 μl of heat-treated cDNA, 5 μl of the Solution II (from the TaKaRa DNA Ligation Kit) and 10 μl of Solution I (from the TaKaRa kit).
4. Incubate the ligation reaction overnight at 16 °C.
5. Stop the reaction by adding 10 μl of H2O, 1 μl of 0.5 M EDTA, 1 μl of 10 % SDS and 1 μl of 20 μg/μl proteinase K. Incubate the solution for 15 minutes at 45 °C.
6. Extract the solution with phenol:chloroform and chloroform, and back-extract with 40 μ1 of H2O.
7. After the ligation, remove the excess linker with S300 spin column chromatography (GE Healthcare Bio-Sciences).
8. Add 4 μl of 5 M NaCl and 160 μl of ethanol (99 %).
9. Centrifuge the mixture at 15,000g for 20 minutes (4 °C) and remove the supernatant. Very gently wash the pellet twice with 500 μl of 80 % ethanol.
10. Dissolve the cDNA in 18.3 μl of H2O.

**Synthesis of second-strand cDNA**

1. Add 0.4 μl of 1 μg/μl T7 promoter primer.
2. Incubate for 10 min at 65 °C and for 5 min on ice.
3. Add:
   - 10X buffer 3 μl
   - 10 mM dNTP solution 1 μl
   - 0.1 M DTT 2.3 μl
   - 3.5 - 4 U/μl DNA polymerase (TaKaRa) 4.5 μl
4. Incubate at 16 °C for 2 hours.
5. Stop the reaction by adding 10 μl of H2O, 1 μl of 0.5 M EDTA, 1 μl of 10 % SDS and 1 μl of 10 μg/μl proteinase K. Incubate the solution for 15 minutes at 45 °C.
6. Extract the solution with phenol:chloroform and chloroform, and back-extract with 40 μ1 of H2O.
7. After the second strand synthesis, remove the excess linker with S200 microspin column (GE Healthcare Bio-Sciences).
8. Add 4 μl of 5.0 M NaCl and 160 μl of ethanol.
9. Centrifuge the mixture at 15,000g for 20 minutes at 4 °C and remove the supernatant. Very gently wash the pellet twice with 500 μl of 80 % ethanol.
10. Dissolve the double-strand DNA in 15 μl of H2O.

**cRNA amplification**

1. Mix following solutions and fill up with H2O to 20 μl finally.
   - 5X Transcription buffer 4 μl
   - 0.1 M DTT 2 μl
   - 100 mM ATP 1.5 μl
   - 100 mM UTP 1.5 μl
   - 100 mM GTP 1.5 μl
   - 100 mM CTP 1.38 μl
   - (CUGA7 in vitro transcription kit from Nippongene)
   - 10 mM cyanine dye CTP (Perkin Elmer) 1.2 μl
   - 0.05 – 0.1 pmol of template DNA 5.92 μl
2. Add 1 μl of CUGA T7 RNA polymerase (Nippongene).
3. Incubate for 2 hours at 37 °C.
4. Purify the sample with RNeasy Kit (Qiagen) according to the manual of Agilent.

**Hybridization and scanning**

The hybridization and scanning procedures are performed according to the Agilent’s manual (<http://www.chem.agilent.com/>).
